# Overview on Chikungunya Virus Infection: From Epidemiology to State-of-the-Art Experimental Models

**DOI:** 10.3389/fmicb.2021.744164

**Published:** 2021-10-05

**Authors:** Larissa E. C. Constant, Bia F. Rajsfus, Pedro H. Carneiro, Tháyna Sisnande, Ronaldo Mohana-Borges, Diego Allonso

**Affiliations:** ^1^Departamento de Biotecnologia Farmacêutica, Faculdade de Farmácia, Universidade Federal do Rio de Janeiro, Rio de Janeiro, Brazil; ^2^Laboratório de Biotecnologia e Bioengenharia Estrutural, Instituto de Biofísica Carlos Chagas Filho, Universidade Federal do Rio de Janeiro, Rio de Janeiro, Brazil

**Keywords:** chikungunya virus, epidemiology, pathogenesis, *in vitro* cell model, rodent models, non-human primate models, cell entry, replicative cycle

## Abstract

Chikungunya virus (CHIKV) is currently one of the most relevant arboviruses to public health. It is a member of the *Togaviridae* family and *alphavirus* genus and causes an arthritogenic disease known as chikungunya fever (CHIKF). It is characterized by a multifaceted disease, which is distinguished from other arbovirus infections by the intense and debilitating arthralgia that can last for months or years in some individuals. Despite the great social and economic burden caused by CHIKV infection, there is no vaccine or specific antiviral drugs currently available. Recent outbreaks have shown a change in the severity profile of the disease in which atypical and severe manifestation lead to hundreds of deaths, reinforcing the necessity to understand the replication and pathogenesis processes. CHIKF is a complex disease resultant from the infection of a plethora of cell types. Although there are several *in vivo* models for studying CHIKV infection, none of them reproduces integrally the disease signature observed in humans, which is a challenge for vaccine and drug development. Therefore, understanding the potentials and limitations of the state-of-the-art experimental models is imperative to advance in the field. In this context, the present review outlines the present knowledge on CHIKV epidemiology, replication, pathogenesis, and immunity and also brings a critical perspective on the current *in vitro* and *in vivo* state-of-the-art experimental models of CHIKF.

## Introduction

Chikungunya virus (CHIKV) is an arthritogenic arbovirus from *Togaviridae* family and *Alphavirus* genus, which is responsible for recurring epidemics over the years worldwide (Mason and Haddow, 1957). CHIKV is considered an important public health problem because it is endemic in tropical and subtropical regions of the globe. Its transmission occurs by the bite of infected mosquitoes from genus *Aedes spp.*, mainly *Aedes aegypti* and *Aedes albopictus*, which are highly domesticated and extremely adaptable to environment changes, respectively, thereby resulting in an efficient spread across the countries and continents (Azevedo et al., 2015; World Health Organization, 2017).

Chikungunya virus infection results in a disease known as Chikungunya fever (CHIKF), characterized by high fever, rash, myalgia, headache, and a prominent polyarthralgia (Burt et al., 2017). Indeed, the name “Chikungunya”, which means “to become contorted” in the Kimakonde language, reflects the most remarkable characteristic of this disease, which is the intense and persistent joint pain (Mavalankar et al., 2008). This symptom is present in more than 90% of the symptomatic cases and can last for weeks, months, or even years in some individuals after complete virus clearance, resulting in a notorious economic and social impact (Wahid et al., 2017; Suhrbier, 2019). Although CHIKF is known as a non-deadly disease, atypical and severe acute manifestations can evolve to multiple organ failure and death. Mortality rates can range from 0.024 up to 0.7% and seem to depend on both the virus genotype/strain and the commitment of neurological system (Jaffar-Bandjee et al., 2010; de Brito, 2017; Dorléans et al., 2018; Freitas et al., 2018; da Silva et al., 2018; Suhrbier, 2019).

Despite the relevance of CHIKV infection to public health, there is still no vaccine or an effective antiviral drug for either the prevention or treatment of CHIKF. In contrast to other arboviruses, such as Dengue (DENV) and Zika (ZIKV) viruses, in which validated and trustable experimental models are largely known and used, experimental models for studying CHIKV infection are diverse and not rarely reproduce just a piece of the pathogenesis observed in humans, which is a challenge for vaccine and drug development. Therefore, understanding the whole picture of this complex disease as well as the current laboratory limitations is vital to address the major issues involving CHIKV infection. In the present review, we outline the major points of CHIKV epidemiology, replication, and pathogenesis and also address the current *in vitro* and *in vivo* state-of-the-art experimental models of chikungunya.

## Epidemiology and Transmission

The first cases of a chikungunya-like illness in humans were recorded in 1823, in Zanzibar, Africa, followed by the report of a similar epidemic in St. Thomas Island, in the Caribbean, during the years 1827 and 1828 (Halstead, 2015). Since then, no other report of a related disease was noticed until 1952, when episodes of a rheumatic fever affected several people in Tanzania, where, for the first time, CHIKV was isolated, identified, and characterized as an arbovirus. Thus, CHIKV caused sporadic and local outbreaks in Africa and Asia until 2004, when it has spread to approximately 60 countries all over the globe (Schwartz and Albert, 2010), causing large and relevant outbreaks. The most remarkable outbreak occurred between 2005 and 2006 in La Reunion Islands in the Indian Ocean. During this epidemic, over one-third of the island’s population was infected, around 260,000 people, with an average of 40,000 new cases per week and 284 deaths (Josseran et al., 2006). In addition, it was the first evidence that a new vector specie, the mosquito *A. albopictus*, actively contributed to virus propagation (Thiboutot et al., 2010). As this mosquito is highly adaptable to temperate zones, a reflect of this outbreak was the identification of the first autochthonous outbreak in Europe 2 years later (in Italy in 2007) (Halstead, 2015). Although the entrance of CHIKV in the Americas had probably occurred by the same time, CHIKF was only reported in the Americas in 2013 (Morens and Fauci, 2014), when the first CHIKV outbreak occurred in Saint Martin, with 658 confirmed cases and an infection rate of 1.76% (Henry et al., 2017). Since then, local transmission has been identified in approximately 45 countries and territories in the Americas, resulting in more than 3 million confirmed cases (Jain et al., 2008; Yactayo et al., 2016; da Cunha and Trinta, 2017; Wahid et al., 2017).

Different CHIKV genotypes have been identified since its discovery: Asian, the East Indian (IOL), the West Africa (WA), and the East/Central/South Africa (ECSA) (Nunes et al., 2015). The ECSA and WA genotypes are endemic in sub-Saharan Africa causing intermittent outbreaks, whereas the Asian genotypes are more restricted to Southeast Asia (da Cunha and Trinta, 2017). The IOL was first identified in 2004 as a descendant lineage from ECSA, and it was responsible for the epidemics that occurred in the Indian Ocean islands and Asia between 2005 and 2011 (Nunes et al., 2015).

At least two CHIKV interconnected transmission pathways take place: the sylvatic and the urban cycles. In the first, CHIKV is maintained in a sylvatic transmission cycle between forest dwelling *Aedes* mosquitoes and non-human primates resulting in sporadic human cases and small outbreaks (Diallo et al., 1999; Petersen et al., 2010). The other is the most relevant to public health and occurs by cyclic transmission of CHIKV from infected to non-infected individuals by the aid of *A. aegypti* and *A. albopictus* mosquitoes, the most relevant vectors of the urban cycle (Jain et al., 2008; Wahid et al., 2017). In this scenario, an adaptive Ala-Val mutation at position 226 in the E1 protein gene (E1:A226V) of an ECSA lineage strain abolished virus dependence on cholesterol to replicate, enhancing not only its infectivity but also CHIKV transmission by *A. albopictus*, which was crucial for virus spread to different continents (Kumar et al., 2008; Azevedo et al., 2015; Madariaga et al., 2016).

In addition to the classical sylvatic and urban transmission cycles, CHIKV infection can also occur by vertical transmission during pregnancy and blood transfusion. Despite not being the most relevant transmission paths, it comes to attention the ability of CHIKV to explore new routes, which is, by itself, a signal of alert for uncontrolled transmission and potential risk of pandemics (Madariaga et al., 2016). Vertical transmission was observed all over the pregnancy stages, but the effects of CHIKV infection in neonates are diverse, varying from asymptomatic to severe, in which myocarditis and/or meningoencephalitis are the most relevant signs of severity (Cardona-Correa et al., 2017). The literature indicates an increased risk for development of severe symptoms in neonates if mother is under viremia period during the childbirth but this risk softens if the infection occurs at least 4 weeks prior to birth (Robillard et al., 2006; Farias et al., 2019). Appassakij et al. (2020) reported that individuals infected with CHIKV can also be potential disease spreaders through blood transfusions or transplants, especially during an outbreak period. This event was observed during the outbreaks occurred in La Reunion, Italy, Thailand, and Puerto Rico. The prevalence of CHIKV RNA in blood donations ranged from approximately 0.4–2.1% during the epidemics. Therefore, an extra care should be taken during the transfusion processes in places where CHIKV is endemic or when outbreaks are ongoing (Petersen et al., 2010; Appassakij et al., 2013, 2020; Petersen and Epstein, 2014; Stanley et al., 2021).

## Clinical Aspects and Pathogenesis

Incubation of CHIKV in humans varies from 1 to 12 days (Panning et al., 2008; Kam et al., 2009; Burt et al., 2012), and viremia can reach up to 3.3 x 10^9^ copies/ml in the first week of infection (Parola et al., 2006; Simon et al., 2007; Panning et al., 2008; Appassakij et al., 2013). It notably contrasts to other arboviruses, mainly from *Flaviviridae* family, such as DENV and ZIKV, from which highest viremia levels varies between 10^4^ and 10^6^ copies/ml in the same period of infection (Srikiatkhachorn et al., 2012; Valiant et al., 2019). Despite most of CHIKV-infected individuals are symptomatic, less than 15% of infected population do not develop any symptoms (Burt et al., 2017).

Chikungunya fever is a spectrum of disease characterized by high, persistent, and self-limited fever, headache, myalgia, and moderate to severe polyarthralgia (da Cunha and Trinta, 2017). Serological exams from CHIKF patients indicate lymphopenia and/or moderate thrombocytopenia and high levels of alanine transaminase (ALT), aspartate aminotransferase (AST), creatinine, and creatinine kinase, which demonstrate the commitment of the liver and kidneys in the infection. In some individuals, calcium deficiency might also happen, which could be related with the cases in which bone absorption occurs (Thiberville et al., 2013; Bedoui et al., 2018). The symptoms usually disappear between the first- and second-week post-infection, occurring together with the restoration of serological parameters. Nevertheless, 30–40% of the cases evolve to a chronic phase, in which debilitating arthralgia persists for months or even years (Borgherini et al., 2008; Schwartz and Albert, 2010; Marimoutou et al., 2012; Schilte et al., 2013). The genetic and immunological factors that drive the chronicity of arthritic symptoms are still not understood. Dermatological manifestations can also occur in 40–50% of the infected population, usually appearing after the beginning of classical symptoms, between the second and the fifth days, and persist for at least 2 days (Borgherini et al., 2007; Inamadar et al., 2008). These manifestations are characterized by skin rashes in the face and the limbs, facial edema, and oral mucosa bleeding (Burt et al., 2012; Caglioti et al., 2013). In adults, the incidence of atypical and severe cases, which are usually associated to hospitalization, increases with age and elderly people are more prone to develop severe manifestations. Respiratory complications, high blood pressure, and cardiac problems are one of the main complications associated to CHIKF severity. Notwithstanding, newborns are the most susceptible to it. Transmission from mother to fetus occurs at the time of birth in the case of intrapartum maternal viremia. Infected neonates usually develop pain, prostration, fever, and thrombocytopenia within few days after birth, and some of them may even have encephalopathy and intracranial bleeding with persistent sequelae (Gérardin et al., 2008; Ramful et al., 2014). In addition to age, personal lifestyle is also correlated to a poorest prognosis of the disease, as previously demonstrated that excessive alcohol ingestion increases mortality rates by CHIKV (da Cunha and Trinta, 2017).

Although CHIKV is markedly an arthritogenic virus, it can also infect the nervous system. Among neurological complications, the most prevalent symptoms seem to be abnormal mental status, headache, focal deficits, and seizures. Other symptoms such as meningoencephalitis, meningoencephalomyeloradiculitis, myeloradiculitis, myelitis, myeloneuropathy, external ophthalmoplegia, facial palsy, sensorineural deafness, and optic neuritis were described during the recent epidemics (Pinheiro et al., 2016). In addition, it was detected the virus RNA in the eye tissue, which correlates to the manifestation of papillitis, retrobulbar neuritis, and neuroretinitis (Mahendradas et al., 2010; Couderc et al., 2012). Encephalitis occurs either simultaneously or within a few days after the onset of systemic symptoms, during the viremia period (Caglioti et al., 2013; Madariaga et al., 2016; Pinheiro et al., 2016). Guillain-Barre syndrome as well as mild hemorrhage, myocarditis, and hepatitis were also reported and are usually observed in both the elderly population and individuals with comorbidities (Lemant et al., 2008; Lebrun et al., 2009; Agarwal et al., 2017; Silva and Dermody, 2017). Alves-Leon and colleagues demonstrated that CHIKV patients with inflammatory demyelinating disease have genotypic resemblance with neurological autoimmune diseases patients, as multiple sclerosis (MS) and neuromyelitis optica spectrum disorders (NMOSD) (Alves-Leon et al., 2021).

Chikungunya virus is usually considered a non-life-threatening disease, but fatal cases have been described. In addition to the most relevant CHIKV outbreak which occurred in La Reunion islands that resulted in 284 deaths (Josseran et al., 2006), an outbreak in Brazil led to 68 fatal cases from which CHIKV RNA was detected in cerebrospinal fluid of at least 92.3% of them, according to the Brazilian Ministry of Health. These data clearly bring an important conclusion that CHIKV neurological commitment is a severity factor directly correlated to the mortality rate. In addition, virus lineage also correlates with mortality being the ECSA lineage the most relevant as it was detected in most of the fatal cases (de Lima et al., 2020).

## Chikungunya Virus, Cell Entry, and Replicative Cycle

Chikungunya virus is a spherical and enveloped virus with an approximately 70 nm of diameter (Silva and Dermody, 2017). Its genome consists of a single-strand positive-sense 12 kb-long RNA with two open reading frames (ORFs) separated by a non-codifying junction and two non-translated regions named 5′UTR and 3′UTR. The 5′ORF is translated from the genomic RNA (gRNA) and codifies the non-structural polyprotein (P1234) that will be further cleaved in individual non-structural proteins nsP1 to 4. The 3′ORF is translated from a positive-sense subgenomic mRNA (sgRNA) and codifies the structural proteins: capsid (C), envelope 3 (E3), envelope 2 (E2), 6K, and envelope 1 (E1) (Strauss and Strauss, 1994; Khan et al., 2002; Solignat et al., 2009; Silva and Dermody, 2017). The role of each of the CHIKV proteins is summarized in Table 1.

**TABLE 1:** Characteristics of CHIKV structural and non-structural proteins.

Protein	Length (aa)	Functions and characteristics	Function by domain	Post-translational modification	References
nsP1	535	• Membrane anchor for replication complex • Capping viral RNA • Association with lipid-rafts • Affinity with cholesterol	• N-terminal: methyltransferase (MTase) and guanylyltransferase (GTase) • Intermediary: membrane binding domain (MB) • C-terminal: D3 domain	Palmitoylated	Lampio et al., 2000; Rana et al., 2014; Feibelman et al., 2018; Zhang N. et al., 2019; Bakhache et al., 2020; Gottipati et al., 2020
nsP2	798	• Essential for capping process • Nonstructural polyprotein cleavage • Shut-off host transcription and translation • Localized in both cytoplasm and cell nucleus • Repression of host antiviral response	• N-terminal: helicase, nucleoside-triphosphatase (NTPase) and RNA-triphosphatase (RTPase) • C-terminal: cysteine protease and methyltransferase-like	Gluthathionylated	Peränen et al., 1990; Pastorino et al., 2008; Karpe et al., 2011; Das et al., 2014; Rana et al., 2014; Stapleford et al., 2015; Rausalu et al., 2016; Saisawang et al., 2017; Göertz et al., 2018; Meshram et al., 2019
nsP3	530	• Contribution to viral genome replication • Contribution to vírus assembly • Binding to ADP-ribose • ADP-ribosylhydrolase activity • Interaction with host factors	• N-terminal: macrodomain • Intermediary: alphavirus unique domain (AUD) • C-terminal: hypervariable domain	Phosphorylated	Li et al., 1990; Vihinen et al., 2001; Dé et al., 2003; Fros et al., 2012; McPherson et al., 2017; Remenyi et al., 2017; Agback et al., 2019; Gao et al., 2019; Shimizu et al., 2020
nsP4	611	• Responsible for viral RNA synthesis • Terminal adenylyltransferase (TdT) activity	• N-terminal: disordered region • C-terminal: RNA-dependent RNA polymerase (RdRp)		Tomar et al., 2006; Rubach et al., 2009; Rupp et al., 2011; Rathore et al., 2013, 2014; Chen et al., 2017
C	261	• Nucleocapsid assembly • Initiation of virus budding process • Self-cleavage • Cytoplasm localization • Contains nuclear localization signals (NLS)	• N-terminal: RNA binding domain • C-terminal: serine protease domain		Hong et al., 2006; Thomas et al., 2010, 2013; Sharma et al., 2018
pE2 (or p62)	487	• E2-E3 precursor • Cleaved by host furin • Contains signal peptide sequence for transportation of nsP1234 to ER		Glycosylated	Strauss and Strauss, 1994; Singh A. et al., 2018
E1	439	• Major envelope protein • Interaction with E2 to form spike-like structure • Contains the fusion loop • Target of neutralizing antibodies	• Domain I • Domain II: type II fusion class • Domain III: Ig-like domain • Transmembrane domain: type I integral membrane	Glycosylated	Metz et al., 2011; Sánchez-San Martín et al., 2013; Masrinoul et al., 2014
E2	423	• Major envelope protein • Interaction with E1 to form spike-like structure • Main target of neutralizing antibodies	• Domain A: receptor binding • Domain B: receptor binding • Domain C • Subdomain D: stem region	Glycosylated	Glasgow et al., 1991; Metz et al., 2011; Silva et al., 2014; Weber et al., 2017; Holmes et al., 2020; Kumar et al., 2020
E3	64	• E1-p62 heterodimer synthesis control • Prevention of premature fusion of E1-E2 with host membrane • Protection of fusion loop at E1 domain		Glycosylated	Singh A. et al., 2018
6K	61	• Signal peptide for E1 • Present in virus envelope • Assistance of E1 translocation to ER • Ion channel activity • Important to virus budding		Glycosylated	Snyder et al., 2013; Silva and Dermody, 2017; Singh A. et al., 2018; Dey et al., 2019
TF	76	• Ion channel activity • Associated to virus production, pathogenesis and budding		Palmitoylated	Snyder et al., 2013; Silva and Dermody, 2017; Singh A. et al., 2018; Dey et al., 2019

**FIGURE 1:**
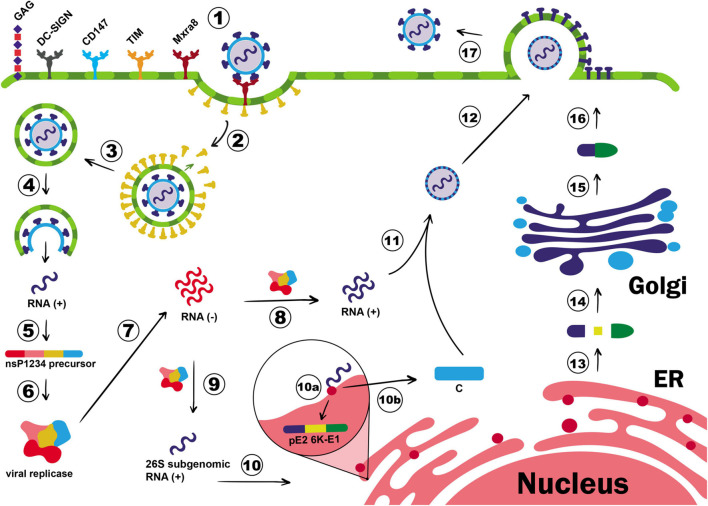
Chikungunya virus (CHIKV) cell entry and replication. E2 glycoprotein binds to the membrane receptor Mxra8, inducing the translocation of clathrin molecules to the plasma membrane (1). GAG, DC-SIGN, CD147, and TIM are also described as CHIKV co-receptors, but their role for entry process is not well elucidated. CHIKV entry occurs *via* clathrin-mediated endocytosis pathway (2) and once the early endosome is formed, clathrin molecules dissociate from the endocytic vesicle (3), and the endosome proceed in the endocytic pathway. The pH acidification of endocytic vesicles triggers the detachment of E1-E2 heterodimers, exposing the fusion loop, which will culminate in the fusion of the endosomal with the viral membranes (4). Then, the nucleocapsid is released in the cytoplasm, genomic RNA is exposed, and translation of the non-structural polyprotein P1234 will take place (5). The P1234 protein is thus cleaved by the viral protease nsP2, releasing the individual non-structural proteins, which will form the viral replicase complex (6). The replicase complex is responsible for the synthesis of the negative-strand RNA (7) that will be the template for new positive-strand RNA (8) as well as for the synthesis of 26S subgenomic RNA (9). The subgenomic RNA, in its turn, is translated into the structural polyprotein C-pE2-6K-E1 in the rough endoplasmic reticulum (RER) (10). The C protein, which contains a protease domain responsible for its self-cleavage, dissociates from the polyprotein just after its translation (10b) and will attach to the positive polarity genomic RNA to form the nucleocapsid in the cytoplasm (11). In this meantime, the pE2-6K-E1 precursor will be addressed to the lumen of the ER (10a), where its maturation process will take place (13). The structural proteins will proceed in the exocytic pathway (14), until the end of E1-E2 heterodimers is mature (15). E1-E2 dimers will be deposited in the cell membrane forming the ‘virus budding microdomain’, a membrane domain where the budding process will occur (16). The recently assembled nucleocapsid migrates to this region, and new virions will be released to the extracellular milieu by budding (17).

After CHIKV is inoculated to the host organism, E2 glycoprotein binds to the membrane receptor Mxra8 on the target cells, which activate an internal signaling pathway resulting in the commitment of clathrin molecules to the plasma membrane and CHIKV clathrin-mediated endocytosis (Sourisseau et al., 2007; Bernard et al., 2010; McMahon and Boucrot, 2011; van Duijl-Richter et al., 2015; Zhang et al., 2018; Basore et al., 2019; Song et al., 2019; Figure 1). Following this event, clathrin molecules are separated from the endocytic vesicle and the acidification of endosomal pH triggers the detachment of E1-E2 heterodimers. This protein rearrangement results in the exposition of the fusion loop, a small motif of 19 residues on E1 protein, that drives the fusion of the endosomal with the viral membranes (Voss et al., 2010; Fields and Kielian, 2013). In addition to the key role of E1 and E2 in the recognition of target cell and membrane fusion process, Ooi et al. (2013) identified two other membrane proteins, which are the fuzzy homologue protein (FUZ) and the tetraspanin membrane protein (TSPAN9), required for the proper infection process by using a genome-wide small interference RNA (siRNA). The FUZ is involved in the clathrin-mediated endocytosis pathway, and TSPAN9 helps viral entry by two possible mechanisms: (i) virus orientation to the early endosome and/or (ii) modulation of the endosome membrane to be more permissive to the fusion process (Ooi et al., 2013). Nevertheless, other molecules, such as glycosaminoglycans (GAGs), T-cell immunoglobulin and mucin (TIM) family, Dendritic Cell-Specific Intercellular adhesion molecule-3-Grabbing Non-integrin (DC-SIGN), AXL receptor tyrosine kinase, and membrane protein complex CD147, have all been described to participate in CHIKV-target cell interaction and to act as alternative cell receptors for CHIKV (Silva et al., 2014; Acharya et al., 2015; van Duijl-Richter et al., 2015; Schnierle, 2019; McAllister et al., 2020; De Caluwé et al., 2021), although binding to them might not be sufficient to trigger virus internalization (Wang et al., 1992; Klimstra et al., 2003; La Linn et al., 2005; Kielian et al., 2010). Other infection routes occur in epidermal and muscle cells. In the first cell line, CHIKV enters the cell by epidermal growth factor receptor substrate 15 (Eps15)-dependent pathway, and in the second cell line, micropinocytosis seems to be the preferred path, which shows the adaptive evolution of CHIKV to infect host cells by several means other than clathrin-dependent endocytosis (Bernard et al., 2010; Lee et al., 2019).

After the membrane fusion, the virus nucleocapsid is released to the cytoplasm, where protein C quickly detaches from the gRNA, which is immediately translated into the non-structural polyprotein P1234. The polyprotein is further cleaved by the viral protease nsP2 at the nsP3/4 cleavage site, releasing the viral polymerase nsP4 and the polyprotein P123 (Shin et al., 2012). The nsP4 will thus synthesize the negative-strand RNA used as a template for new copies of positive-polarity RNA (Figure 1). It is believed that the synthesis of the positive-strand is connected to the processing of the P123, as its cleavage into individual proteins maintains the synthesis of the positive-strand RNA but interferes in the minus-strand RNA synthesis (Strauss and Strauss, 1994). Indeed, P123 processing is associated with the sgRNA formation, as this event redirects the replication complex toward vesicular cytoplasmic spherules that will host double strand viral RNA (dsvRNA), protecting it from degradation and/or recognition by intracellular dsRNA sensors (Silva and Dermody, 2017). The polyprotein P123 is then cleaved at nsP1/2 followed by processing the nsP2/3 site. Interestingly, some CHIKV isolates encode an opal stop codon located after nsP3, which can control the expression of nsP4, by a read-through mechanism (Solignat et al., 2009). After these events, all individual nsPs are produced and the synthesis of the structural proteins is initiated.

The 3′ORF of the sgRNA is translated into a structural polyprotein that is further cleaved into individual proteins by viral and host proteases. Structural proteins are required for many viral processes, including virus assembly, receptor binding, and membrane fusion (Jose et al., 2009). The first produced structural protein is the C, a multifunctional protein responsible for packaging viral RNA and drive virion budding process (Hong et al., 2006; Thomas et al., 2010). The C protein contains a serine-protease domain responsible for its self-cleavage from the rest of the structural polyprotein (Thomas et al., 2010). The ability to exert a proteolytic activity indicates the relevance of this protein for successful new virus production, as its cleavage does not depend on host machinery. In other words, the rate of C protein production is the determinant step for new virus assembly. Once produced, C protein oligomerizes and opsonizes the gRNA to form the nucleocapsid core (Sharma et al., 2018; Figure 1).

During CHIKV replication, the ratio of gRNA:sgRNAs can vary between 1:3.5 to 1:5.5 (Scholte et al., 2013). Therefore, the success of virus assembly depends on the ability of C protein to distinguish between them and also other host’s small RNAs. It was believed that the presence of a packaging signal (PS) composed by structural RNA elements located in the nsP2 gene was the key element responsible for it (Kim et al., 2011). However, it was recently discovered that both CHIKV and Semliki Forest virus (SFV) do not exclusively depend on the PS at nsP2 to assertively package the gRNA. In fact, the first 2/3 of gRNA contains several binding sites for C protein, and its interaction in these regions drives proper RNA selection and nucleocapsid assembly (Brown et al., 2020).

Following this event, the structural polyprotein pE2-6K-E1 is conducted to the endoplasmic reticulum (ER) and Golgi apparatus, on account of a signal peptide sequence present in its N-terminal region (Firth et al., 2008; Snyder et al., 2013), where they will be processed and post-translational modifications, such as glycosylation, will take place. Host proteases, such as furin, cleave the structural polyprotein into individual E1, E2, E3, and 6K, which are further used for building the new virion particles (Yap et al., 2017). A ribosomal frameshift in the translation of the 6K gene might happen resulting in the production of the Transframe protein (TF) that shares the same N-terminal domain of 6K but different C-terminal and is involved in viral production, pathogenesis, and virus budding processes (Snyder et al., 2013; De Caluwé et al., 2021). During the exocytic pathway, post-translational modifications on E1 and E2 glycoproteins allow their association in heterodimers complexes, composing the virus envelope (Yap et al., 2017). Succeeding these events, the nucleocapsid core moves to membrane regions rich in E1-E2 dimers and mature virion is released by budding process from the infected cell (Figure 1). The complete budding mechanism is still not completely understood, but some interesting studies have reported the dependence of optimal temperature and pH conditions, as well as the presence of host cell membrane cholesterol to occur (Marquardt et al., 1993; Lu and Kielian, 2000; Lu et al., 2001). In addition, viral release is intensified by the presence of 6K and TF proteins, since the deletion or mutation in their genes negatively modulates the rate and the efficiency of virion budding, indicating their relevance to the process (Gaedigk-Nitschko and Schlesinger, 1991; Lu and Kielian, 2000; Ramsey and Mukhopadhyay, 2017).

## Cell and Tissue Tropism

Chikungunya virus can bind to several cellular receptors and undergo different internalization pathways. It exhibits wide cell, tissue, organ, and organism tropism, and the understanding of where and how the infection occurs in each site of active replication is the first step in the fight against this virus.

In invertebrate hosts, several tissues are susceptible to CHIKV and infection occurs very quickly. The midgut epithelium appears to be the first site of viral replication (Monteiro et al., 2019) followed by propagation to secondary organs, such as the salivary glands (Wong et al., 2016). By the way, infection of this tissue is the key step to make the mosquito a competent vector, since the transmission occurs when it salivates during blood feeding and the released saliva contains infectious CHIKV particles. The time between feeding with infected blood and the ability to transmit to vertebrate hosts, known as extrinsic incubation period (EIP), is a valuable parameter to estimate transmission rate and viral load during feeding. In case of CHIKV, the EIP can be as short as 2 days and quantification of viral RNA can be as high as 10^4.8^ PFU in salivary gland and 10^3.3^ PFU in extracted saliva (Dubrulle et al., 2009).

In humans and non-human primates, CHIKV primarily targets epithelial tissue in the area of inoculation. Epithelial fibroblast, keratinocytes, and melanocytes are susceptible to CHIKV (Sourisseau et al., 2007; Puiprom et al., 2013; Ekchariyawat et al., 2015; Gasque and Jaffar-Bandjee, 2015; Wichit et al., 2017; Matusali et al., 2019). Indeed, infection of these cells is a key step for stablishing the disease since CHIKV titer is rapidly increased, which is essential to reach other targets without being completely neutralized by host immune system. However, it is still unknown whether infection of epithelial cells exert any other effect in the pathogenesis than just an internal virus reservoir. After reaching the bloodstream and lymphatic system, CHIKV will infect blood cells and other tropism organs, such as liver, joints, muscles, brain, and spleen (Ozden et al., 2007; Her et al., 2010; Ruiz Silva et al., 2016; Suhrbier, 2019; Tritsch et al., 2020). Blood monocytes, B lymphocytes, and plasmacytoid dendritic cells (pDCs) are susceptible to CHIKV infection (Hawman et al., 2016; Ruiz Silva et al., 2016; Webster et al., 2018). It can also enter and replicate in synovial and muscles fibroblasts, synovial macrophages, myoblasts, muscle satellite cells, chondrocytes, and osteoblast (Ozden et al., 2007; Hoarau et al., 2010; Chusri et al., 2011; Phuklia et al., 2013; Hussain et al., 2016; Lentscher et al., 2020; Pott et al., 2020). Infection of synovial macrophages is important to keep high viremia during the acute phase (Her et al., 2010; Ruiz Silva et al., 2016; Haist et al., 2017), and viral RNA was detected in these cells in both humans and non-human primates during the chronic phase of the disease, suggesting that persistent viral replication may be related to the maintenance of arthritic symptoms (Hoarau et al., 2010; Labadie et al., 2010; Hawman et al., 2013). It is well known that CHIKV might also infect the nervous system but the mechanism of how the virus cross the blood-brain barrier is still poorly characterized. Endothelial brain cells, neuroblastoma cells, astrocytes, microglial cells, neurons, oligodendrocytes, corneal endothelium, corneal fibroblasts, scleral stroma, ciliary body, iris, and ocular muscle fibers have been reported to be infected by CHIKV but further studies need to be performed to confirm the effect of their infection to CHIKF clinical outcome (Abere et al., 2012; Couderc et al., 2012; Dhanwani et al., 2012; Wikan et al., 2012; Abraham et al., 2013, 2017; Fraisier et al., 2014; Lim and Chu, 2014; Das et al., 2015; Wei Chiam et al., 2015).

## Immunopathogenesis

Chikungunya virus infection is known to cause severe musculoskeletal disorder, but the molecular mechanism involved in this process is not fully understood (Maek-A-Nantawat and Silachamroon, 2009). Observational studies in human subjects revealed that CHIKV infection elicits immune mechanisms similar to autoimmune diseases, which might explain the similarity between the arthritic phenomenon that occurred during the infection with rheumatoid arthritis (RA) (Chirathaworn et al., 2020). We categorize below the innate and the adaptive immune responses during CHIKV infection.

### Innate Immune System

Mosquito saliva has several immunomodulatory molecules in their composition that neutralize the host immune defense to allow an appropriate feeding. CHIKV uses this artifice to hijack host defense and be able to infect target cells on epithelial tissue. On the other hand, after this initial step, CHIKV infection induces an exacerbated local innate immunity (Tanabe et al., 2018; Cook et al., 2019; Foresto et al., 2019; Maucourant et al., 2019; Hiroki et al., 2020) in which macrophages (MØ), natural killer cells (NK), neutrophils, DCs, basophils, and eosinophils are recruited to the site of infection as a result of the release of several chemoattractant molecules by the infected cells (Ng et al., 2009; Waymouth et al., 2013; Chirathaworn et al., 2020). Monocyte chemoattractant protein-1 (MCP-1), granulocyte colony-stimulating factor (G-CSF), and granulocyte-macrophage colony-stimulating factor (GM-CSF) are the main chemokines released by these cells (Couderc et al., 2008; Ng et al., 2009).

Massive monocyte and MØ infiltrate are largely observed in CHIKV infected tissues, including the synovial fluid from chronic CHIKF patients, where it correlates to cartilage and bone destruction (Rulli et al., 2011; Phuklia et al., 2013; Amdekar et al., 2017). MCP-1, as the most active chemoattractant molecule for these cells, plays a key role in the process (Her et al., 2010; Ruiz Silva et al., 2016; Haist et al., 2017). Treatment with bindarit, a MCP-1 inhibitor, resulted in a decrease of inflammatory infiltrate in the joints and muscles in a CHIKV mouse model (Kumar et al., 2012; Nayak et al., 2017; Chirathaworn et al., 2020). Likewise, high levels of Interleukin-1β (IL-1β), Interleukin-6 (IL-6), Interleukin-5 (IL-5), Interleukin-7 (IL-7), Interleukin-10 (IL-10), Interleukin-15 (IL-15), tumor necrosis factor α (TNF-α), C-X-C Motif Chemokine Ligand 9 (CXCL9), C-X-C Motif Chemokine Ligand 10 (CXCL10), Hepatocyte Growth Factor (HGF), Basic Fibroblast Growth Factor (FGF-basic), and Vascular Endothelial Growth Factor (VEGF) are observed in both infected patients and mice models (Ng et al., 2009; Aarreberg et al., 2018). IL-1β produced by CHIKV-infected cells acts primarily as an antiviral molecule being responsible for controlling viral propagation by stimulation of Myeloid differentiation primary response 88 (MyD88) pathway in non-infected cell (Unterholzner and Bowie, 2008; Allen et al., 2009; Ichinohe et al., 2009). MyD88 is an adaptor protein for Toll-like receptors (TLRs) and IL-1β receptor (IL-1βR), and antiviral response occurs by the activation of TLR3/TRIF, TLR7-MyD88, and/or retinoic acid-inducible gene I (RIG-I) pathways (Kozak et al., 1998; Sundgren-Andersson et al., 1998). On the other hand, excessive IL-1β production as well as IL-6 and TNF-α, which are pyretic cytokines, result in an exacerbated pro-inflammatory response that shifts the antiviral response to a robust inflammatory disease. High circulating levels of IL-6 and TNF-α correlate with joint destruction, cellular proliferation and differentiation, and bone absorption, which are observed in both RA and CHIKV infection (La Linn et al., 2005; Yoshida and Tanaka, 2014; Farrugia and Baron, 2016; Goupil et al., 2016). Treatment with immunosuppressive drugs, such as anakira, an IL-1β receptor antagonist, or immune modulators, such as abatacept, a CTLA4 immunoglobulin that binds to CD80/86, resulted in a reduction of inflammatory symptoms and reduced cartilage and bone loss, showing, therefore, the significant role of innate immune response to disease severity (Miner et al., 2017; Wolf et al., 2019).

Interferon (IFN) response is the most relevant antiviral mechanism elicited by host cells to constrain CHIKV replication and propagation (Teng et al., 2015). High levels of circulating IFN-α and IFN-γ were found in both humans and animal models (Schilte et al., 2010; Cook et al., 2019). IFN production is induced after the activation of pattern recognition receptors (PRRs), a group of membrane-associated or intracellular receptors that recognize exogenous molecules, including viral RNA. Released IFNs by infected cells exert an autocrine/paracrine signaling that will activate the Janus Kinase-signal transducer and activator of transcription (JAK-STAT) pathway, through binding to IFN-α/β receptors (IFNAR) (Majoros et al., 2017). Phosphorylated STAT translocates to cell nucleus where it will induce the expression of Interferon-stimulated genes (ISG), which includes pattern-recognition receptors (PRRs), interferon-regulatory factors (IRFs), cytokines and chemokines, and pro-apoptotic molecules. These mediators help non-infected cells to protect themselves against viral infection (Schilte et al., 2010; Wauquier et al., 2011; Simarmata et al., 2016). The whole picture of antiviral mechanisms elicited by host cells against CHIKV is discussed in considerable depth by Nelemans and Kikkert (Nelemans and Kikkert, 2019).

Chikungunya virus evolved interesting mechanisms to block IFN response, most of them mediated by nsP2. Fros and colleagues demonstrated that CHIKV infection resists to the inhibition mediated by IFN and is able to repress IFN activity by negative modulation of ISGs expression. The authors also showed that nsP2 alone can block JAK-STAT signaling pathway (Fros et al., 2010). Nuclear nsP2 promotes the export of STAT1 from nucleus, hampering downstream activation of IFN pathway (Göertz et al., 2018). Moreover, nsP2 together with E2 and E1 act as antagonists of melanoma differentiation-associated gene 5 (MDA5)/RIG-I receptor signaling pathway, directly inhibiting IRF3 and, consequently, the production of IFN-β (Bae et al., 2019). Despite its role against IFN response, nsP2 exerts an additional immune evasion role by shutting off the cellular transcription process through the degradation of the RNA polymerase II catalytic subunit Rpb1(Akhrymuk et al., 2012).

Recently, it was shown that cytosolic DNA sensor cyclic GMP-AMP synthase (cGAS) broadly inhibited RNA viruses and constitutes an addition mechanism to block arbovirus infection (Schoggins et al., 2014; Ahn and Barber, 2019). cGAS induces the dimerization of STING after the detection and binding to foreign DNA or DNA-RNA complexes. STING dimerization activates tank binding kinase 1 (TBK1), which will induce phosphorylation of IRF3 and promote the expression of IFN-I and pro-inflammatory cytokines (Ergun et al., 2019; Motwani et al., 2019). CHIKV can directly antagonize cGAS-STING pathway by degradation of cGAS mediated by C protein (Webb et al., 2020).

### Adaptive Immune System

Albeit the innate immune response can itself eliminate CHIKV, host adaptive immune system is extremely important to complete virus clearance and prevent disease progression (Hoarau et al., 2010; Wauquier et al., 2011). The acute CHIKV infection leads to the activation and proliferation of CD8^+^ T cells, whereas CD4^+^ T response is dominant during the chronic phase (Maek-A-Nantawat and Silachamroon, 2009). Additionally, B and T cell responses might oversee the chronic joint problems due to CHIKV infection (Teo et al., 2012; Poo et al., 2014b). Interestingly, despite the role of cellular immunity to CHIKV infection, an acute lymphopenia during the initial phase of disease is usually observed. The decreased frequency of circulating B and T cells seems to be a transient process, since it is reestablished after this period and probably happens because of their massive migration to infected tissues in first days of infection (Trinchieri, 2010; McCarthy et al., 2018).

Humoral response is also very important to virus depuration. CHIKV structural proteins, especially envelope proteins, are the main targets of neutralizing antibodies. Kym and colleagues analyzed the frequency of anti-CHIKV antibodies produced during the infection, and most of them were against E2, E3, C, and nsP3 proteins (Kam et al., 2012). However, it seems that only anti-E2 antibodies are converted to memory. Neutralizing antibodies constitute the last but a potent strategy to fight against the virus. It acts by at least four different mechanisms: (i) opsonizing virus particle, leading to neutralization of virus entry by hampering the recognition of the target receptor on host cells; (ii) binding to structural proteins in the surface layer of infected cell membrane, inhibiting budding of new virion particles; (iii) eliciting an antibody-dependent cell cytotoxicity (ADCC), in which effector immune cells such as NK and T lymphocytes kill infected cells; and (iv) eliciting antibody-dependent cell phagocytosis (ADCP), in which professional phagocytes, mainly MØ and DC, will clear circulating virus (Jin and Simmons, 2019). Some of these mechanisms have already been described for anti-CHIKV antibodies. Anti-E2 antibodies are able to attenuate the infection by targeting essential epitopes for virus entry and virus release processes (Jin et al., 2015; Tumkosit et al., 2020). These antibodies might also block virus attachment to target cell and suppress membrane fusion process (Pal et al., 2013; Zhou et al., 2020). These findings suggest the potential of anti-CHIKV antibodies as effective prophylactic and therapeutic options against CHIKV infection.

## *In vitro* Cell Models for Studying CHIKV Infection

### Cell Lineages

Chikungunya virus infects and replicates in a plenty of cell types. Cell lineage models are widely used for depicturing CHIKV infection, and this system is regularly used to investigate the entry mechanism, replication cycle, functionality of viral proteins, and the efficiency of antiviral compounds. In this regard, a broad range of cell lineages have been applied to explore these processes and each cell model demonstrates specific outcomes of CHIKV pathogenesis (Table 2).

**TABLE 2:** Summary of primary and immortalized cell lines used in CHIKV studies.

Cell name	Origin
Hs. 789.Sk	Human primary skin fibroblast
MRC5	Human primary lung fibroblast
hSMM	Human primary skeletal muscle myoblast
PBMC	Human primary blood monocytes
FLS	Human primary fibroblast-like synoviocyte
Osteoblasts	Human primary osteoblast
Vero E6	Monkey kidney epithelial-derived cell line
BHK-21	Baby hamster kidney fibroblast-derived cell line
HeLa	Human cervical carcinoma epithelia-derived cell line
HEK-293T	Human embryonic kidney epithelia-derived cell line
293T	Human kidney epithelia-derived cell line
BEAS-2B	Human bronchial epithelia-derived cell line
BGM	Buffalo green monkey kidney-derived cell line
THP-1	Human peripheral blood monocyte-derived cell line
Huh7	Human hepatocellular carcinoma-derived cell line
C6/36	*Aedes albopictus* intestine-derived cell line
A20	Mouse B lymphocyte-derived cell line
AAg2	*Aedes aegypti*-derived cell line
RD	Human rhadbdomyosarcoma-derived cell line
U4.4	*Aedes albopictus*-derived cell line
A549	Human lung adenocarcinoma-derived cell line
U251MG	Human malignant glioblastoma-derived cell line
Vero CCL-81	*Cercopithecus aethiops* kidney-derived cell line
HFF	Human foreskin fibroblast-derived cell line
C2C12	Mouse myoblast-derived cell line
SVG-A	Human astrocyte-derived cell line

Based on a sub-genomic replicon systems and infectious virus, Roberts and colleagues assessed the best physiological cellular model for CHIKV study. Their work suggests that mammalian cell lines Huh7, C2C12, and SVG-A, as well as mosquito cell lines U4.4 and C6/36 are acceptable for *in vitro* infection studies (Roberts et al., 2017). Similarly, Sudeep et al. (2019) evaluated the sensitivity and susceptibility of Vero-E6, BHK-21, RD, A-549, and C6/36 cell lineages to three different CHIKV genotypes. Results demonstrated that Vero-E6, BHK-21, and C6/36 are more susceptible to CHIKV and produced higher viral titer than RD and A-549 cells (Sudeep et al., 2019). C6/36 cell is an admissible *in vitro* model for CHIKV, as it is derived from *A. albopictus* midgut and it is generally used for several arbovirus propagations (Walker et al., 2014; Miller et al., 2018). However, Brackney et al. (2010) reported that this cell line possesses a debilitated RNA interference (RNAi) pathway associated with the cellular antiviral response that hampers studies of mosquito-arbovirus interactions at molecular levels. BHK-21 and Vero-E6 cells are widely used in plaque assays for analysis of viral replication, screening of antiviral compounds, and evaluation of neutralizing antibodies (Franco et al., 2018; Lee et al., 2019; Noval et al., 2019; Sharma et al., 2019; Zhang Y.-N. et al., 2019; de Oliveira et al., 2020; Tumkosit et al., 2020; Pereira et al., 2021). Using a BHK-21 cell model, Santos and colleagues analyzed the potential antiviral properties of the snake venom phospholipase A2_CB_ (PLA2_CB_) on CHIKV replication cycle and demonstrated that this molecule inhibits CHIKV entry process (Santos et al., 2021). Likewise, Singh and colleagues characterized two peptidomimetic compounds as CHIKV protease inhibitors in a BHK-21 cell model and were able to propose their mechanism of action on the replicative process (Singh H. et al., 2018). Vero-E6 was used to study the entry mechanism of a candidate CHIKV vaccine, as well as the inhibition of virus-cell binding in infected cultures treated with CHIKV antibodies (Fritz et al., 2012; Shen et al., 2019; Kiesslich and Kamen, 2020; Weiss et al., 2020). Garg et al. (2020) implemented virus like particles (VLPs) produced in 293T cells as a model for CHIKV vaccines, highlighting a new role of this cell in CHIKV research. HEK-293 is also vastly used in the characterization of viral proteins functions. Saisawang et al. (2017) made a recombinant HEK-293 cell expressing CHIKV nsP2, a model used to identify that this protein is glutathionylated and this modification alters the protease function. In addition, HEK-293 lineage was also the choice model for an interactome study targeting CHIKV nsP3 and nsP4, showing, therefore, that this cell is very useful for CHIKV *in vitro* studies (Rathore et al., 2014). However, regardless of their easiness and wide use, immortalized cell lineages are genetically modified and might not be the best model in some kind of studies. Therefore, use of primary cell lines should be considered based on the research focus.

### Primary Cell Lines

Despite the high cost, short life span, and ethical issues, primary cell lines are categorized as the best *in vitro* models for studies on the alteration of intracellular pathways due to infection. As these cell types are not genetically modified, the obtained results are supposed to be more trustable than those based on cell lineages, although there are some exceptions, as the case of antiviral screening, efficacy of vaccines candidates, and recombinant expression of viral proteins.

As previously mentioned, arthralgia and muscle pain are most characteristic features of CHIKV infection. Therefore, human fibroblast-like synoviocytes (hFLS) and human skeletal muscle myoblast (hSMM) cells are useful in the investigation of altered signaling pathways and their correlation to clinical symptoms. Phuklia and colleagues demonstrated that CHIKV-infected human FLS can release chemokines and differentiation mediators but cannot secrete arthritogenic cytokines. In addition, the supernatants of infected hFLS induced primary human monocyte recruitment and had osteoclastogenic activity (Phuklia et al., 2013). Interestingly, hFLS and hSMM showed altered gene expression patterns associated with interferon production, transcription factors, pro-inflammatory proteins, skeletal and muscular disorders, and virus replication when compared to cell lineages (Hussain et al., 2016; Pott et al., 2020). It was confirmed by a study that identified 26 differentially expressed microRNAs in CHIKV-infected hFLS correlated to the repression of the local immune system and induction of virus persistence (Agrawal et al., 2020). Despite of being the best models to study arthritic phenomenon, obtaining these cells is difficult and tricky. Therefore, other tropism cells can also be used. Blood monocytes are much easier to be obtained, are susceptible to CHIKV, and constitute an excellent model to study the innate immune response against the virus (Her et al., 2010; Aguilar-Briseño et al., 2020). Epithelial fibroblast is also an interesting primary cell line to evaluate first steps of CHIKV infection and the mechanisms that trigger cartilage damage (Ekchariyawat et al., 2015).

## Animal Models for Studying CHIKV Infection

Although cell lines are extremely useful and demonstrated to be a powerful tool to understand the replication and molecular aspects of CHIKV pathogenesis, they may not reflect whole infection scenario. Development of rodent and non-human primate animal models is essential to advance the knowledge on CHIKV pathogenicity as well as to serve as a nonclinical model for anti-CHIKV drug or vaccine development. Table 3 summarizes the state-of-the art animal models used in CHIKV studies.

**TABLE 3:** Summary of *in vivo* rodent and NHP models for studying CHIKV infection.

Category/model	Animal	Main outcome	References
Aged model	WT C57BL/6	Severe disease progression.	Uhrlaub et al., 2016; Arévalo et al., 2019; Jain et al., 2019
Anti-CHIKV treatment	WT C57BL/6, C1q^–/–^, FcRγ^–/–^	Limitation on CHIKV infection.	Abdelnabi et al., 2018; Fox et al., 2019; Patil et al., 2021
Arthritis	WT C57BL/6, ISG15^–/–^, UbE1L^–/–^, MHCII^Δ/Δ^, IFNγ^–/–^, Sting^gt/gt^, CCR2^–/–^	CHIKV arthritis signature.	Gardner et al., 2010; Werneke et al., 2011; Nakaya et al., 2012; Poo et al., 2014a; Geng et al., 2021
CHIK vaccine development	WT C57BL/6; AG129; BALB/c (H2^d^)	Rapid and long-lasting against CHIKV responses. Protection against lethal challenge.	Wang et al., 2011; Arévalo et al., 2019; Campos et al., 2019; López-Camacho et al., 2019; Chen et al., 2020; Adam et al., 2021
Chronic/Persistent model	WT C57BL/6, CD8_α_^–/–^, Batf3^–/–^, Wdfy4^–/–^, Rag1^–/–^, μMT C57BL/6; Golden hamster	Viral persistence in joint tissues. Severe inflammation of the musculoskeletal and joins tissues.	Morrison et al., 2011; Bosco-Lauth et al., 2015; Hawman et al., 2016, 2017; Davenport et al., 2020
IFN receptor–deficient	IFNAR^–/–^, ISG15^–/–^, IFN-α/βR^–/–^	High mortality, paralysis, severe disease.	Couderc et al., 2008; Werneke et al., 2011; Hiroki et al., 2020; McCarthy et al., 2020
Lethal challenge	BALB/c, AG129, DBA1/J, Swiss Webster	Death after 2–13 days post infection*.	ROSS, 1956; Campos et al., 2019; Zhang H.-L. et al., 2019; Chen et al., 2020; Julander et al., 2020
Leukocyte deficient	TLR_3_^–/–^, TLR_3/7/9_^–/–^, TLR_9_^–/–^, CCR2^–/–^	Increased viral load and enhanced disease susceptibility.	Poo et al., 2014a; Hiroki et al., 2020; McCarthy et al., 2020
Acute disease and Innate immune model	Rhesus macaques, Bonnet macaques, Cynomologus macaques	Clinical symptoms, viremia, Immune cells, cytokines, persistence	Chen et al., 2010; Messaoudi et al., 2013; Roy et al., 2014
Aged model	Rhesus macaques	viremia, clinical symptoms and immune response age dependent	Messaoudi et al., 2013
Pregnant model	Rhesus macaques	Viral detection in tissues during pregnancy	Chen et al., 2010
Vaccine and therapies	Rhesus macaques, Cynomologus macaques	Viremia and immune response during therapy	Akahata et al., 2010; Chang et al., 2014; Kam et al., 2014; Pal et al., 2014

### Rodent Model

In general, most of the studies use C57BL/6J wild type (WT) mice and CHIKV inoculation might occur from newborns at just few days after birth up to elderly animals of up to 48 weeks old. Likewise, virus titer can range from 10^2^ to 10^8^ plaque-forming unit (pfu)/ml (Gardner et al., 2010; Bosco-Lauth et al., 2015; Uhrlaub et al., 2016; Abdelnabi et al., 2018; Arévalo et al., 2019; Jain et al., 2019; Adam et al., 2021; Patil et al., 2021). Therefore, it is clear that the existence of a diversity of CHIKV rodent models and not surprisingly divergent outcomes is observed in each of them. One of the pioneering studies on CHIKV mice model was carried out by Ross in 1950’s. He analyzed 6-day-old Albino Swiss animals and observed lethality after intracerebral virus administration. Furthermore, mice resistance to virus challenge was also evaluated in study in which 6–20-day-old mice was infected by the same route and, as result, mice older than 12 days survived to infection (ROSS, 1956), showing a different profile of susceptibility when compared to humans, in which elderly people are more prone to develop the disease.

The main goal in animal models is to replicate most of the disease signature to comprehend the whole pathogenic process as well as the altered physiological and biochemical pathways that contribute to chronicity. In humans, CHIKF induces a broad modification in joints and surrounding tissues physiology (Lam et al., 2001; Byers et al., 2019). In contrast, to obtain these entire outcomes in WT animals is a considerable challenge. Most of reported models reproduce just a part of the disease. For example, a 14-day-old C57BL/6J WT mice inoculated with 10^2^ pfu of CHIKV by a subcutaneous route in the foot developed gross swelling, severe tenosynovitis, and myositis just in the inoculated foot (Morrison et al., 2011). A 3-week-old C57BL/6J mice injected with 10^3^ pfu exhibited higher and longer detectable levels of viral RNA, up to 98 days post infection (dpi) (Abdelnabi et al., 2018). In contrast, after subcutaneous injection of 2 × 10^7^ pfu/ml in 8-week-old C57BL/6J mice in the hind limbs, it was found replicating virus particles only until 9 dpi (Jain et al., 2019). It is possible to observe inflammatory outcomes in an 18-month-old C57BL/6 mice model challenged subcutaneously in the footpad with 10^3^ pfu of CHIKV, in which a prolonged viremia, severe early swelling, and late footpad joint and connective tissue pathology were detected (Uhrlaub et al., 2016). On the other hand, other group described that the same mice strain with up to 12 weeks old can develop CHIKV resistance (Arévalo et al., 2019). Circulating virus was observed until 15 dpi in 20-week-old C57BL/6 mice challenged with 2 × 10^7^ pfu/ml by subcutaneous route in the hind limbs (Jain et al., 2019). In an attempt to reproduce arthritic symptoms, Gardner and colleagues inoculated 1 × 10^6^ pfu of CHIKV in 4-week-old C57BL/6 mice by intramuscular route and observed that animals developed muscle degeneration, atrophy, marrow mononuclear cell infiltration, and edema. In the same study, the authors showed that 6-week-old mice inoculated with CHIKV subcutaneously were also able to develop rheumatic symptoms (Gardner et al., 2010).

Julander and colleagues explored the effects of different CHIKV lineages on different mice strains. Using 4–6-week-old DBA/1J and AG129 mice, they infected them with 10^4.5^ or 10^7.5^ cell cytotoxic infectious dose 50 (CCID)/ml and 10^1.5^ or 10^2.5^ CCID50/0.1 ml, respectively, *via* subcutaneous route in the footpad and hocked of the right leg. They observed a virus strain-dependent pathogenesis, being the strains from IOL and WA clades more virulent than the others (Julander et al., 2020). Bosco-Lauth et al. evaluated 4–6-week-old and 6-month-old golden hamsters infected by intraperitoneal route. Animals developed inflammatory lesions on skeletal muscle, fascia, and tendon sheaths of multiple limbs (Bosco-Lauth et al., 2015).

Despite the complexity of CHIKV infection, animal model is important and indispensable. WT mice lineages present a complete genetic and metabolic background allowing the investigation of diseases without depletion of one or more signaling pathways, which can hijack the translation to human disease. However, considering the complexity of CHIKF, the use of WT mice constitutes inherent obstacles on data consistence. Therefore, transgenic mice popped up as a useful tool to figure out the mechanism of CHIKF pathogenicity.

### Genetically Modified Rodent Models

Single-gene-knockout animals have been developed in an attempt to understand the contribution of a specific component or pathway for a disease or condition establishment. In the case of CHIKV infection, these transgenic mice seem to be more susceptible to develop a human-like disease constituting, therefore, a valuable tool for either assessing disease pathogenesis or screening new vaccines and antiviral compounds. As example, 3-week-old C1q^–/–^ or FcRγ^–/–^ C57BL/6J mice seemed to be an immunocompetent mouse model for studying CHIKV-induced arthritis (Fox et al., 2019). The ISG15^–/–^ mouse, in its turn, exhibited increased susceptibility to viral infection (Morales et al., 2015). Double knockout (dKO) UbE1L^–/–^ and ISG15^–/–^ C57BL/6J mice with 6 and 9 days old infected with CHIKV exhibited increased levels of pro-inflammatory cytokines and chemokines, correlating to human cytokine and chemokine profile, and also showed increased lethality rate to viral infection (Werneke et al., 2011). Upregulation of genes associated with activation of macrophages, activation, and movement of phagocytes were observed in mutant MHCII^Δ/Δ^ and IFNγ^–/–^ mice inoculated with CHIKV 10^8^ pfu by subcutaneous route toward the ankle (Nakaya et al., 2012). Infection with 3 × 10^5^ pfu CHIKV in the hind footpad of Sting-deficient mice (Sting^gt/gt^) of 6–12 weeks old resulted an increase of immune cells in the muscle/synovial cavity/tendon compared to WT group. Interestingly, Sting is apparently a nonessential pathway for the IFN-α response during CHIKV infection mice (Geng et al., 2021). Infected Rag1 KO exhibited a persistence of virus on joint-associated tissues. In addition, C57BL/6 μMT mice were unable to control CHIKV infection (Hawman et al., 2016). Also, 3–5-week-old congenic Rag1^–/–^ and Irf3^–/–^ Irf7^–/–^ dKO inoculated in the left footpad with 10^3^ pfu of CHIKV developed a disease independent of Irf3-, Irf7-, and IFNAR1-antivirals response pathway (Hawman et al., 2017). IFN-α/βR^–/–^ mice showed increased susceptibility to CHIKV infections despite otherwise preserved immune responses (Müller et al., 1994). Hiroki and colleagues evaluated the neutrophil extracellular traps during CHIKV infection in TLR3^–/–^, TLR3/7/9^–/–^ (triple knockout), TLR9^–/–^, and IFNAR^–/–^ C57BL/6 or 129S6/SVEV background mice (Hiroki et al., 2020). Neutrophil extracellular traps (NETs), which are a component of the innate immune response, protected the animals against infection, showing a central role in immune defense against the virus (Papayannopoulos, 2018). They found that NET release occurs through a TLR7- and ROS-dependent mechanism during CHIKV infection (Hiroki et al., 2020). Pregnant IFN-α/βR^–/–^ mice at 16–18 days of gestation were infected with 20 pfu of CHIKV *via* the intradermal route. As result, placenta viral titers were at least 2 orders of magnitude lower and fetuses were not infected, which conflicts with what is observed in humans (Ramful et al., 2007; Couderc et al., 2008; Cardona-Correa et al., 2017; Di Maio Ferreira et al., 2019). Overall, the use of transgenic mice for studying CHIKV revealed an interesting option and some models were able to reproduce most of the symptoms and characteristics of human disease, although the entire outcome was still not being achieved.

### Non-human Primate (NHP) Models

Non-human primates (NHPs) is also a regularly used animal model for CHIKV research. This model provides key advantages for studying different aspects of CHIKV disease compared to murine models as their physiology is closer to humans and they developed classical clinical symptoms of CHIKF. The first CHIKV NHP experiments were performed using Rhesus macaques (*Macaca mulatta*) in 1960’s, demonstrating that these animals were able to produce neutralizing antibodies when inoculated with viremic human sera, and also developed clinical symptoms of CHIKF, including fever (Binn et al., 1967). Along with Rhesus macaques, bonnet macaques (*Macaca radiata*) and cynomolgus macaques (*Macaca fascicularis*) are also used to depicture CHIKV pathogenesis, being good models to assess the influence of age (Messaoudi et al., 2013) and pregnancy (Chen et al., 2010) as well as to screen vaccines (Akahata et al., 2010) and immunotherapeutic candidates (Kam et al., 2014).

Rhesus and cynomolgus macaques infected with CHIKV had detectable viremia for at least 6 days, with peak levels 1–2 dpi (Akahata et al., 2010; Chen et al., 2010; Messaoudi et al., 2013; Pal et al., 2014). Also during the first week of infection, the animals developed high fever and rash (Chen et al., 2010), correlating to human disease evolution. Right after the infection, frequency of innate immune cells in the peripheral blood of infected animals revealed an increase in monocytes/macrophages and all dendritic cell subset. After 10–14 dpi, T-cell and B-cell proliferative responses reached its peak. CHIKV-specific Ab response reaches its plateau around 21 dpi, and second burst of memory B-cell proliferation occurs only at 28 dpi (Messaoudi et al., 2013). A decrease of immune response against pathogens has been associated with aging in NHP, highlighting its usefulness to study aging impact of CHIKV infection. A CHIKV-infected 17-year-old rhesus macaques showed significant differences in viremia, clinical symptoms, and the CHIKV-specific immune response compared to adult rhesus macaques with 6–13 years old. Based on this study, immune senescence is suggested to be a key factor in CHIKV disease severity (Messaoudi et al., 2013). A pregnant rhesus macaque model of 7–15 years old at gestational days 121–132 was used to assess CHIKV infection during pregnancy (Chen et al., 2010). Similar to what is observed in non-pregnant animals, viremia level peaked at 2–3 dpi, and appearance of fever and changes in blood cell counts correlated with peak viremia. Joint swelling was developed just in a limited number of animals, and viral RNA was detectable in the spleen and lymph nodes of the pregnant macaques 21 dpi. Although viral RNA was present in several maternal tissues, fetal tissues and placenta demonstrated no histological changes or virus presence (Chen et al., 2010). These results contrast with the data of human intrauterine CHIKV infections (Ramful et al., 2007; Cardona-Correa et al., 2017).

Regarding the screening of vaccines and immunotherapies candidates, NHP reveals to be a good model because of their similarities with human’s physiology and pathogenesis of CHIKV infection. All CHIKV vaccine technologies developed so far have been tested in NHP. The immunization of rhesus macaques with attenuated CHIKV resulted in a reduced viremia and induction of anti-CHIKV antibody production by the day 14. This model was also used to evaluate the efficacy of two live-attenuated CHIKV-IRES vaccine candidates, several subunit vaccine candidates, and a CHIKV virus-like particle candidate (Akahata et al., 2010; Labadie et al., 2010; Mallilankaraman et al., 2011; Kam et al., 2014). The results showed similar endpoints than those observed in human trials, corroborating the relevance of these models for drug and vaccine development. Likewise, the efficacy of monoclonal humanized antibodies against E1 and E2 proteins was tested in rhesus macaques and the treatment demonstrated clear protection against CHIKV infection. This treatment resulted in no viremia at 2 dpi and reduced RNA load in the tissues (Pal et al., 2014).

Although these models have inherent difficulties, such as harder ethical issues and difficult and expensive maintenance cost, their potential as a preclinical model for testing therapeutics and vaccines are clearly consolidated. The main advantage of NHP is the similar pathogenesis and immunological response to CHIKV to that observed in humans. Therefore, the use of NHP is an interesting choice regarding drug/vaccine development.

## Conclusion

Chikungunya virus have recently become an urgent problem to public health because of several reasons, such as (i) the large and important outbreaks occurred in the last decade resulting in thousands of hospital interventions and hundreds of deaths, (ii) its possible widespread all over the globe as the result of virus ability to propagate in different vector species, and (iii) the high rate of long-term debilitating arthralgia with direct and significant social and economic impact. Several contributions have been made to understand the mechanisms associated to virus replication and pathogenesis. We know now that a plethora of cell types are susceptible to CHIKV infection, some of them directly contributing to both the establishment and maintenance of the disease, some of them acting to prevent the evolution of viral infection, whereas others have a minor but not less import role, serving as virus reservoir. Understanding the most affected tissues and cell types allows the development of reproducible, validated, and robust cellular and animal models to study CHIKV infection in the pursuit of helping the development of therapeutic and vaccine options to manage CHIKF. In this regard, our current knowledge is that CHIKF is a very complex disease in which it is impossible to reproduce the full disease signature in a unique *in vivo* model. Instead, there are several approaches and transgenic models that reproduce pieces of the disease and together might contribute to disease comprehension and to serve in development of antiviral technologies.

## Author Contributions

LC, BR, PC, TS, and DA drafted, revised, and prepared the manuscript. TS and RM-B revised the manuscript. RM-B and DA conceptualized and made the final revision on the manuscript. DA finalized the manuscript for submission. All authors contributed to the article and approved the submitted version.

## Conflict of Interest

The authors declare that the research was conducted in the absence of any commercial or financial relationships that could be construed as a potential conflict of interest.

## Publisher’s Note

All claims expressed in this article are solely those of the authors and do not necessarily represent those of their affiliated organizations, or those of the publisher, the editors and the reviewers. Any product that may be evaluated in this article, or claim that may be made by its manufacturer, is not guaranteed or endorsed by the publisher.
